# Apoptosis-inducing factor and calpain upregulation in glutamate-induced injury of rat spiral ganglion neurons

**DOI:** 10.3892/mmr.2015.3626

**Published:** 2015-04-16

**Authors:** ZHONG-JIA DING, XIN CHEN, XIAO-XU TANG, XI WANG, YONG-LI SONG, XIAO-DONG CHEN, JIAN WANG, REN-FENG WANG, WEN-JUAN MI, FU-QUAN CHEN, JIAN-HUA QIU

**Affiliations:** 1 Department of Otolaryngology-Head and Neck Surgery, Xijing Hospital, Fourth Military Medical University, Xi’an, Shaanxi 710032, P.R. China; 2 Outpatient Department, Logistics Academy, Beijing 100858, P.R. China

**Keywords:** glutamate, perfusion, culture, cochlea, spiral ganglion neurons, apoptosis-inducing factor, calpain

## Abstract

Spiral ganglion neuron (SGN) damage and apoptosis can lead to noise-induced hearing loss, age-associated hearing loss and, in certain cases, auditory neuropathy. The apoptosis-inducing factor (AIF)-associated pathway may be important in this process. The present study aimed to investigate the expression levels of AIF and calpain in damaged SGNs. Glutamate (Glu) perfusion and cell culture in different concentrations of Glu were performed to damage the SGNs of Sprague-Dawley (SD) rats, with saline water used as a control Different concentrations (5, 10, 20 and 40 mM) of Glu were injected into the cochlear tympanic canal of 18 SD rats, and 10, 20 and 40 mM Glu were added to SGN cultures. Auditory brainstem responses (ABR) were measured prior to and 2 days following the injection of Glu. Immunofluorescent staining was used to detect the SGN damage and the expression levels of AIF and calpain *in vivo* and in *in vitro*. Transmission electron microscopy (TEM) was used to measure cell apoptosis and reverse transcription-quantitative polymerase chain reaction was used to analyse the gene expression levels of AIF and calpain in the damaged SGNs. The TEM identified mitochondrial vacuolisation, swelling of the SGN and hetero-chromatin formation. Injection of Glu reduced the number of SGNs and induced apoptosis. AIF was observed to translocate into the nuclei of the SGNs in the 20 and 40 mM Glu groups, and the expression levels of AIF and calpain were markedly upregulated in the modiolus of the Glu-damaged SGNs. The upregulation of AIF and calpain may be important in the process of SGN damage and apoptosis.

## Introduction

Apoptosis-inducing factor (AIF) is a flavoprotein, which usually performs a redox reaction in the electron transport chain (1) and induces apoptosis under conditions of injury. AIF is cleaved by calpain is and released from the mitochondria into the cytoplasm, where it relocates to the nucleus, cleaves DNA into fragments and induces apoptosis (2,3). AIF is commonly expressed in cells, however, apoptosis is not induced in all types of cell (4), only in neuronal cells and certain tumour cells. Calpains belong to a family of calcium-dependent, non-lysosomal cysteine proteases, which are expressed ubiquitously in all cells (5). Regulated by the amount of Ca^2+^, calpain has been previously reported to promote apoptosis (5,6). Apoptosis is central in neural injury disease, including spiral ganglion neuron (SGN) injury (7). SGNs, as the first afferent neuron in the auditory pathway, have become a focus of investigation. SGNs are susceptible to damage and the induction of apoptosis by noise, ischemia and hypoxia, which can lead to noise-induced hearing loss, age-associated hearing loss and even auditory neuropathy (8,9).

In the cochlea, SGN damage and apoptosis are regulated by a complex signaling pathway. Fu *et al* perfused ouabain into the cochlea to induce SGN apoptosis via the expression of caspase (9). Schmutzhard demonstrated that severe sepsis-induced hearing loss can be attributed to apoptosis of the supporting cells, mediated by an upregulation of caspase 3 (10). However, inhibition of caspases results in incomplete or limited protection, and AIF is concentrated in cochlea sensory cells. A previous study observed that 122 dB white noise induces the relocation of AIF into the nuclei (11).

Therefore, in the present study, Glu was directly perfused into the cochlear tympanic canal (12) and SGNs were cultured *in vitro* in order to examine the expression levels of AIF and calpain in Glu-damaged SGNs.

## Materials and methods

### Animals and ethical statement

A total of 18 female Sprague-Dawley rats, aged 4–6 weeks and weighing between 150 and 180 g, were used for liquid perfusion, and postnatal 0–3-day old rats were used for the *in vitro* culture of SGNs. The rats were maintained in clean conditions, with *ad libitum* access to food and water at 37°C and 40% humidity. All animals were provided by and cared for by the Institutional Animal Care and Use Committee of the Fourth Military Medical University (Xi’an, China). The present study was approved by the Ethics Committee of Xijing Hospital (Xi’an, China).

### Liquid perfusion

The rats for the liquid perfusion analysis were administered 2% sodium pentobarbital (0.25 ml/100 g; Sigma-Aldrich, St. Louis, MO, USA) to induce anaesthesia, and placed on a board at 37°C to maintain their temperature. The skin and fascia surrounding the ear were dissected, and the muscles were separated to expose the otocyst. A power drill (XiChang Inc., Xi’an, China) was used to drill a hole in the otocyst, revealing the cochlea and the artery at the top of the hole. A hole (~0.2 mm in diameter) was then opened on the wall of the scala tympani using a hand drill (XiChang Inc.). A plastic tube connected to a Hamilton syringe pump (XiChang Inc.) was used to inject different concentrations of Glu (Hexin Chemical Industry, Shenzhen, China) for 30 min. Subsequently, the wound was closed. All the animals were divided into six groups, which received either injected saline water (control), 5, 10, 20 or 40 mM glutamate, or no treatment. In the control animals, the normal saline was perfused into the perilymph via the same procedure and in all rats, with the exception of the untreated rats, surgery was performed on both ears.

### Auditory brainstem response (ABR)

The auditory thresholds were determined by measuring the ABR. A TDT III System Auditory Evoked Potential Workstation, controlled using SigGenRZ and BioSigRZ software (Tucker-Davis Technologies, Fort Lauderdale, FL, USA) was used to collect data. The ABRs were elicited using tone bursts (4, 8, 16, 24 and 32 kHz; 0.5 ms rise/fall time; no plateau; alternating phase). The stimulus was provided through an RZ6 D/A converter (Tucker-Davis Technologies) and was presented using a high-frequency speaker (MF1 Multi-Field Magnetic Speakers; Tucker-Davis Technologies) placed ~2 cm in front of the ear being assessed. The stimulus was reduced in 5 dB steps until the response ceased. The differential potential was sampled over 10 ms, filtered (low-pass, 4 kHz; high-pass, 100 Hz) and averaged (512 sweeps of alternating stimulus polarity) in order to obtain the mean traces at each intensity. The rats were assessed 2 days following the perfusion, and the resulting data were analysed using SPSS version 17.0 software (SPSS, Inc., Chicago, IL, USA).

### SGNs culture

Dissociated spiral ganglion cultures were prepared, as described previously, from postnatal 0–3 day rats (13). In brief, the SGNs (~1×10^4^) were incubated in SGN culture medium: Dulbecco’s modified Eagle’s medium with B27 (2 ml/ml; Sigma-Aldrich), brain-derived neurotrophic factor (10 *µ*g/ml; Sigma-Aldrich), penicillin (100,000 U/l; 1%; Sigma-Aldrich). Subsets of the cultures were maintained at 37°C in a humidified incubator (Heraeus CO_2_; Bole Company, Beijing, China) with 5% CO_2_, which was divided into four groups, in which either 10, 20 or 40 mM Glu was added to cells for 48 h at 37°C.

### Immunofluorescent staining

The slides were fixed by perfusing with 4% paraformaldehyde (XiChang Inc.) for 1 day, following which, the slides were immersed in 0.1 M phophate-buffered saline (PBS; Huamei Biotech Co., Ltd.) for 20 min and Triton X-100 (0.3%; Sigma-Aldrich) for 15 min. The slides were washed with 0.1 M PBS for 5 min each time and were then incubated at 37°C in a 5% goat-serum blocking solution (Huamei Biotech Co., Ltd., Wuhan, China) for 30 min. The slides were incubated with primary antibodies against AIF (polyclonal goat; 1:100; Santa Cruz Biotechnology, Inc., Dallas, TX, USA) and β-tubulin (poly-clonal rabbit; 1:200; Abcam, Cambridge, MA, USA) at 4°C in a refrigerator for 3 days. The slides were then washed with 0.1 M PBS and were incubated with Alexa Fluor 488 (poly-clonal donkey anti-goat; 1:200; Invitrogen Life Technologies, Carlsbad, CA, USA) and Cy3 (polyclonal goat anti-rabbit; 1:100; Abcam) at 4°C for 1 day. Subsequent to the addition of 0.1% DAPI (Sigma-Aldrich) to stain the nuclei, glycerol (Huamei Biotech Co., Ltd.) was used to seal the coverslips. The sections were observed using a laser scanning confocal microscope (FV1000; Olympus Corporation, Tokyo, Japan).

### Transmission electron microscopy (TEM)

The treated rats were fixed using a 2.5% glutaraldehyde (Huamei Biotech Co., Ltd.) solution. The rats were sacrificed by decapitation, and the cochlea was removed from the head and immersed in the same fixative at 4°C overnight. The specimens were then rinsed with 0.1 M PBS and immersed in a 10% EDTA (Sigma-Aldrich) solution for 7 days for decalcification. Subsequently, the cochlea was placed in a 1% osmic acid solution (Abcam) for 2 h at 4°C. The entire cochlea was removed from the bony shell, embedded and cut into ultrathin transverse sections (70–100 nm) using an ultramicrotome (Reichert UltracutE, Leica, USA). Counterstaining was then performed using uranyl acetate (Huamei Biotech Co., Ltd.) for 50 min and lead citrate (Huamei Biotech Co., Ltd.) for 10 min. Finally, the specimens were observed using a TEM (JEM-1230; Olympus Corporation).

### Reverse transcription-quantitative polymerase chain reaction (RT-qPCR)

The RNA from the treated cochleae were used for RT-qPCR, according to the QuantiTect Reverse Transcription (Qiagen, Valencia, CA, USA) instructions. RT-qPCR analysis was performed using a SYBR^®^ Green Master Mix kit (Applied Biosystems Life Technologies, Foster City, CA, USA) and RNase-free 96-well PCR plates. The qPCR was performed on a CFX96 Touch™ Real-Time PCR Detection system (Bio-Rad Laboratories, Inc., Hercules, CA, USA). The following forward and reverse primers (Genecopoeia, Rockford, MD, USA) were used for the specific RNAs in RT-qPCR: R-AIF, forward 5′-TAGAACTCCAGATGGCAAGACA-3′; R-AIF, reverse 5′-AAGCCCACAATAAGGACTAACAC-3′; R-caspase 3, forward 5′-GAATGACTGGGAGTGGGGTAG-3′; R-caspase 3, reverse 5′-GACCTGGAACATCGGATTTGA-3′; R-calpain, forward 5′-CAAAGTGGACCCCTATGAACG-3′; R-calpain, reverse 5′-TAAGGGCGTCAGGTGTAAGGT-3′. The threshold cycles (Ct) value of the genes under examination in each sample were normalised using the value of the endogenous control gene, 18S. The relative fold changes in gene expression levels were obtained by comparing the 2^-ΔΔCt^ data of the different groups.

### Data analysis

Wherever possible, the data are expressed as the mean ± standard deviation. Statistical significance was determined using analysis of variance followed by a multiple comparison Dunnett’s test (using SPSS software). P<0.05 was considered to indicate a statistically significant difference.

## Results

### ABR thresholds increase following Glu perfusion

**Figure 1:**
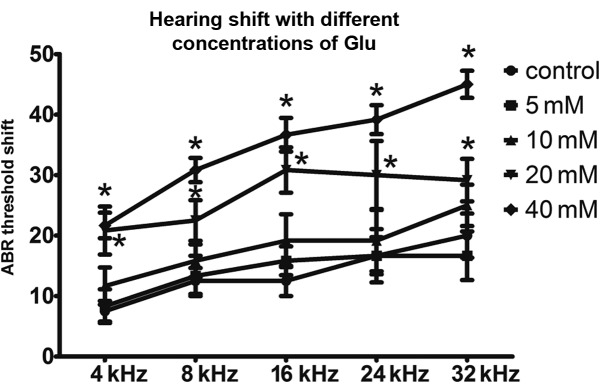
Hearing shift data subsequent to the perfusion with different concentrations of Glu. The average ABR thresholds were increased at 4, 8, 16, 24 and 32 kHz. No significant differences were observed between the control group and the 5 and 10 mM groups, however, significant increases were observed in the 20 and 40 mM groups. Higher concentrations of Glu perfusion were associated with higher shifts in frequencies. (^*^P<0.05, vs. 5 mM, 10 mM and control groups). Data are expressed as the mean ± standard deviation. Glu, glutamate; ABR, auditory brainstem responses.

Subsequent to Glu perfusion, the ABR threshold was markedly increased. No significant difference was observed among the values for the control, 5 or 10 mM Glu groups (P>0.05), however, a significant increase was observed in the 20 and 40 mM Glu groups (P<0.05). In the control and 5 mM groups, there was a 7–8 dB shift at 4 kHz, in the 10 mM group, the shift reached 11 dB, and in the 20 and 40 mM groups, the shift reached 20 dB. At 8 and 16 kHz, the shift in the control and 5 mM groups was 12–15 dB, however, there was a shift of 20–30 dB in the 10, 20 and 40 mM groups. At 24 and 32 kHz, the shift was <20 dB in the control, 5 and 10 mM groups, but was >20 dB in the other groups. The ABR shifts increased with the frequency of the toneburst (Fig. 1).

### Glutamate toxicity reduces the number of cochlear SGNs and alters their morphology

**Figure 2:**
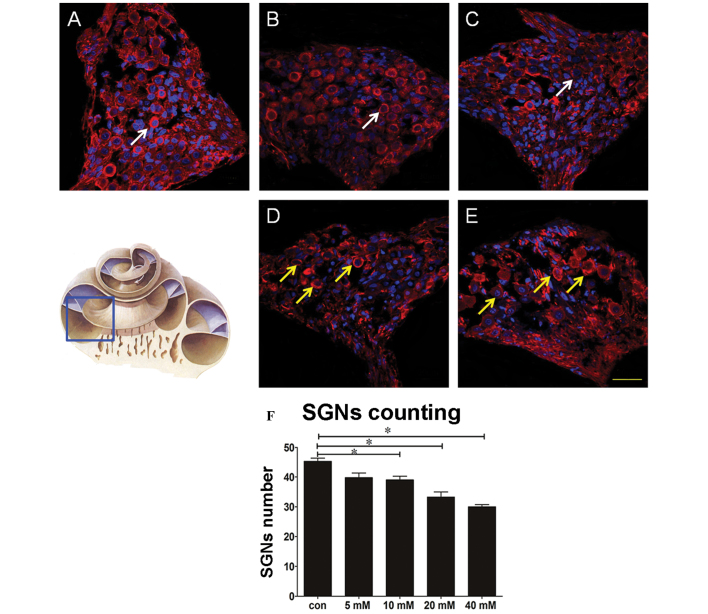
Morphology of the SGNs and the proportion of remaining SGNs compared with the control group. Tubulin (red) indicates the morphology of the SGNs, and DAPI (blue) indicates the nuclei. Immunofluorescence images in the (A) control group and at Glu perfusion concentrations of (B) 5, (C) 10, (D) 20 and (E) 40 mM. (F) Histogram depicting the proportion of SGNs lost in each image. White arrows indicate normal SGN; yellow arrows indicate abnormal SGN or irregular morphology. Scale bar, 50 *µ*m. Data are expressed as the mean ± standard deviation. SGNs, spiral ganglion neurons; Glu, glutamate; con, control.

**Figure 3:**
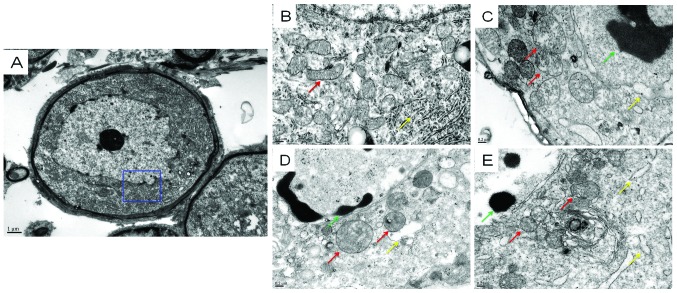
Morphology of SGNs in the Glu perfusion groups via transmission electron microscopy. Images of the SGNs in the (A) control cells and at Glu perfusion concentrations of (B) 5, (C) 10, (D) 20 and (E) 40 mM (magnification, x40,000). Yellow arrows indicate the endoplasmic reticulum, red arrows indicate the mitochondria and green arrows indicate the heterochromatin gathered around the nuclear membrane. Swelling and vacuoles were observed in (C), (D) and (E). Scale bar, 50 *µ*m. SGNs, spiral ganglion neurons; Glu, glutamate.

A total of three successive sections of the cochlea middle axis were selected for tubulin staining. Images were captured using a laser scanning confocal microscope and the mean number of basal SGNs present in each group were counted by two or more individuals. The Glu perfusion groups were compared with the control, providing the rates of SGN loss. The results demonstrated that the mean number of SGNs was reduced by a magnitude of between two and three in the 5 and 10 mM Glu groups, compared with the control group, and by 8–9-fold in the 20 and 40 mM Glu groups (data not shown). The statistical analyses revealed that the numbers of SGNs in the latter groups were significantly lower compared with those in the that of the 5 and 10 mM Glu groups (Fig. 2). TEM also revealed that treatment with Glu at ≥10 mM generated a high number of cytoplasmic vacuoles and a significant quantity of heterochromatin around the nuclei in the SGNs, in addition to swelling of the mitochondria and endoplasmic reticulum (Fig. 3). It was, therefore, concluded that excessive Glu damaged the SGNs in the cochlea, in a dose-dependent manner.

### Expression levels of caspase 3 are unchanged, whereas those of AIF and calpain are altered in Glu toxicity-induced SGNs

**Figure 4:**
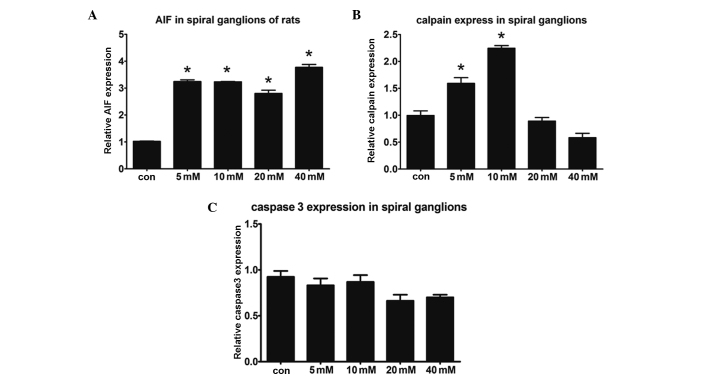
Reverse transcription-quantitative polymerase chain reaction results for AIF, caspase 3 and calpain following Glu perfusion. The expression levels of (A) AIF, (B) calpain and (C) caspase 3 in all the treatment groups are shown. The AIF histogram reveals upregulation of AIF in all the Glu perfusion groups, compared with the control group. The calpain histogram reveals significant upregulation in the 5 mM and 10 mM groups, compared with the other groups. (^*^P<0.05). No significant differences were observed between the expression levels of caspase 3 in the Glu perfusion groups and in the control group. The ΔΔCt method was used to measure the mRNA transcription levels in the SGNs. Data are expressed as the mean ± standard deviation. AIF, apoptosis-inducing factor; Glu, glutamate; con, control; SGNs, spiral ganglion neurons.

The perfused rats were decapitated on a clean bench, Rosenthal’s canal and the middle axis were separated and the basilar membrane and spiral ligament were removed. RT-qPCR was performed to detect the expression levels of AIF, calpain and caspase 3. Increased expression levels of AIF were observed in the Glu intervention groups, whereas the expression levels of caspase 3 were not significantly different. Thus, SGN damage did not upregulate the expression of caspase 3. Changes in the expression levels of calpain were also observed in all the treatment groups. In the 5 and 10 mM Glu groups, the expression levels of calpain were significantly higher compared with those of the 20, 40 mM and control groups (P<0.05; Fig. 4). In conclusion, AIF, rather than caspase 3, initiated the Glu toxicity-induced SGN apoptosis. Calpain was also involved in the damage of the SGNs.

### AIF is translocated into the nuclei following exposure to Glu at concentrations ≥20 mM

**Figure 5:**
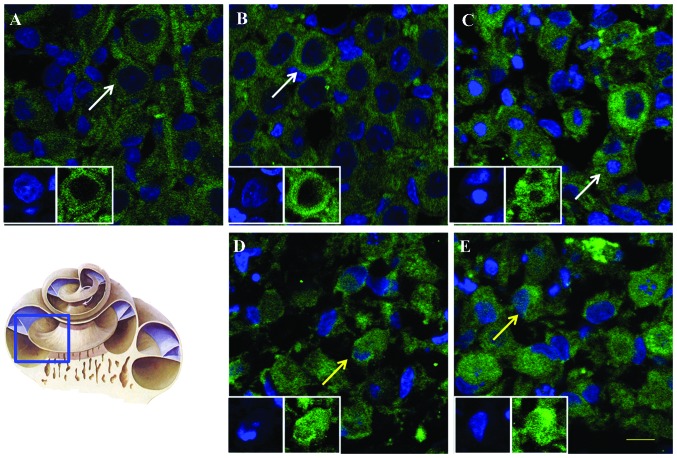
Immunofluorescence results of the distribution of AIF in SGNs perfused with Glu. Immunofluorescence images at in the (A) control, and at Glu perfusion concentrations of (B) 5, (C) 10, (D) 20 and (E) 40 mM. Scale bar, 50 *µ*m. AIF (green) is located in the cytoplasm in the normal group, whereas the nuclei (blue) are visible in the central regions of the SGNs. In the 10 mM, 20 mM and 40 mM groups, the green and blue signals overlap. White arrows indicate cells, in which AIF was distributed in the cytoplasm, and yellow arrows indicate cells, in which AIF overlapped with the nuclei. AIF, apoptosis-inducing factor; SGNs, spiral ganglion neurons; Glu, glutamate.

To assess the localisation and expression levels of AIF, the subcellular location of AIF was examined in SGNs following Glu perfusion. The results demonstrated that, in the groups treated with ≥20 mM Glu, the red fluorescence of AIF overlapped with the blue nuclear staining in the laser scanning confocal microscopy; however, concentric circles of red labelling surrounded the blue labelling were observed in the control group. The staining patterns of the perfused groups were irregular and included incomplete nuclear staining and polygonal cytoplasmic labelling. These altered patterns of expression suggested that the AIF was initally translocated into the nuclei of the SGNs (Fig. 5).

### AIF is translocated into the nucleus and the expression levels of AIF and calpain, but not caspase 3 are upregulated in Glu-treated SGNs in vitro

**Figure 6:**
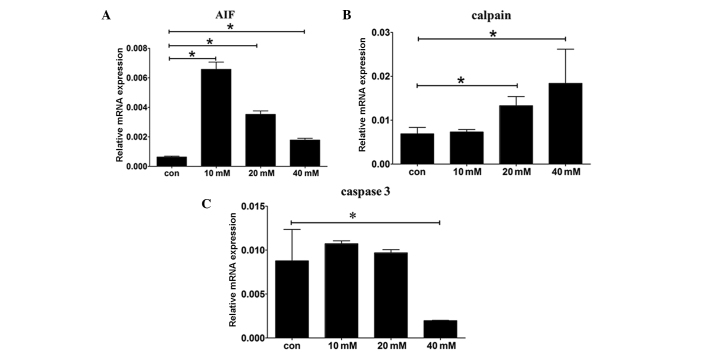
Reverse transcription-quantitative polymerase chain reaction results of AIF, caspase 3 and calpain in cultured SGNs. (A) mRNA expression of AIF was increased following Glu intervention, with significant increases in expression observed in the 10, 20 and 40 mM groups (P<0.05 vs. con). (B) mRNA expression of calpain was also upregulated, with significant increases in the 20 and 40 mM groups (P<0.05 vs. con). (C) Expression of caspase 3 was unhanged following intervention, whereas the expression in the 40 mM group was significantly reduced (P<0.05 vs. con). Data are expressed as the mean ± standard deviation. AIF, apoptosis-inducing factor; SGNs, spiral ganglion neurons.

To confirm the mRNA expression levels of AIF and calpain, the SGNs were incubated in medium, containing different concentrations of Glu. The mRNA expression levels of AIF and calpain increased following the addition of Glu for 48 h, and the expression levels of AIF were significantly higher, compared with the control, in the 10, 20 and 40 mM groups (P<0.05). The expression levels of calpain were also higher in the 20 and 40 mM groups compared with the control (P<0.05). However, the expression levels of caspase 3 were not significantly different in the four groups (P>0.05). These results demonstrated that AIF and calpain were upregulated in Glu-treated SGNs *in vitro*, however, the levels of caspase 3 were unchanged (Fig. 6).

**Figure 7:**
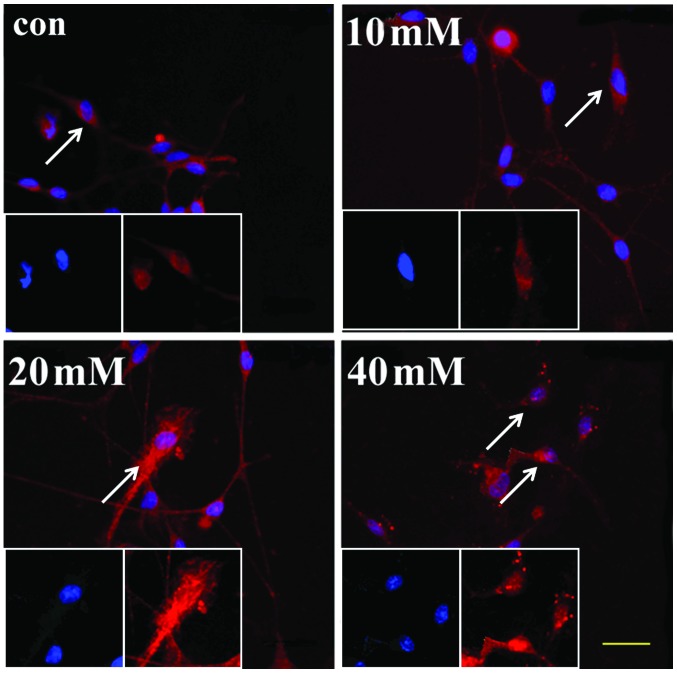
Immunofluorescence results of the distribution of AIF in cultured SGNs. AIF was stained red and the nuclei were stained blue. In the control and 10 mM groups, AIF was distributed in the cytoplasm without overlap of the blue nuclei. However, AIF overlapped with nuclei in the 20 mM group (white arrow), which demonstrated that AIF had translocated into the nuclei. In the 40 mM group, the levels of AIF in the nuclei were increased. AIF, apoptosis-inducing factor; SGNs, spiral ganglion neurons.

To observe the distribution of AIF, an immunostaining assay was performed. In the control group, the red-stained AIF was distributed in the cytoplasm only. However, in the 20 mM group, AIF had translocated into the nuclei of certain bipolar neurons, which was observed in greater number of cells in the 40 mM group (Fig. 7). In conclusion, the addition of a high concentration of Glu induced the translocation of AIF into the nuclei, and the upregulation of AIF and calpain in the cultured SGNs.

## Discussion

Spiral ganglia, as the first afferent nerves, transmit acoustic information to the auditory centre, by which they are regulated (8). Excessive Glu has been reported to damage SGNs, inducing sensorineural hearing loss and auditory neuropathy (13). Auditory neuropathy is a disease, in which the inner hair cells fail to transmit acoustic information to the brain. This disorder clinically presents with elevated hearing thresholds without a parallel loss of otoacoustic emissions, for which damage to the SGNs may be the cause (3). Increased Glu binds with Glu receptors, including NMDAR2 and mGluRIs, and initiates calcium regulation and oxidative damage, injuring the SGNs (14), which leads to subsequent hearing loss. In the present study, ABR threshold shifts increased with increasing concentrations of perfused Glu. The 20 and 40 mM Glu groups had a greater shift, between 4 kHz and 32 kHz, indicating damage mediated by Glu. The number of SGNs was significantly reduced with increasing concentrations of Glu, and TEM identified heterochromatin surrounding the nuclei, which confirmed the damage to the SGNs by excessive Glu and demonstrated a successful model of SGN-damage. These results provide support in further examining the mechanisms of auditory neuropathy. Investigating higher concentrations of Glu may complete experiment data and provide clearer presentation of Glu-damaged SGNs.

Caspase has been a focus of early investigations of cochlea sensory cells. Noise and ototoxic drugs can induce caspase upregulation in outer hair cells, however, Steinbach and Lutz observed that addition of the Z-VAD-FMK caspase inhibitor to SGN cultures prevents the apoptosis of SGNs (14). Abaamrane *et al* injected Z-VAD-FMK into the cochlea, which resulted in incomplete recovery in deafness, following detonation (15). In the present study, qPCR revealed that the levels of caspase 3 were not upregulated in response to Glu perfusion or SGN culture, which supported the hypothesis that the caspase pathway has a limited effect on Glu toxicity in SGNs (14). However, the RT-qPCR results demonstrated that the mRNA expression levels of AIF increased following Glu treatment, compared with the control group, and the immunostaining assay indicated that AIF was translocated into the nuclei at Glu concentrations of ≥20 mM perfusion, in the perfusion and cultured SGNs. These results demonstrated that AIF may be induced in SGN apoptosis and contribute to Glu-mediated SGN injury in sensory hearing loss and auditory neuropathy.

Calpain has been identified as being important in the association between Glu toxicity and AIF in apoptosis (16). Vosler *et al* demonstrated that calpain can be activated by apoptotic signals, releasing AIF from the inner mitochondrial membrane into the cytoplasm (17), while other studies have observed that AIFis then transferred between the cytoplasm and the nucleus to induce nuclear DNA fragmentation (3,18), which results in an influx of Ca^2+^ into the cytoplasm through the ion channels of the NMDA Glu receptors and the activation of calpain. In the RT-qPCR results of the present study, the expression of calpain was upregulated subsequent to Glu acting on the SGNs via Glu perfusion or following the addition of Glu to cultured SGNs, highlighting the important role of calpain. TEM demonstrated morphological alterations of the endoplasmic reticulum in the SGNs, and suggested that endogenous Ca^2+^ was involved in calpain activation in the cytoplasm (19). The results suggested that the calpain-AIF upregulation pathway was important in Glu-mediated damage of SGNs.

In conclusion, the present study demonstrated an explanation for the mechanism underlying Glu-mediated SGN damage in tympanic canal perfusion and *in vitro* SGNs intervention. This may assist in understanding the relevant mechanisms of auditory neuropathy.
